# Acupuncture therapy: mechanism of action, efficacy, and safety: a potential intervention for psychogenic disorders?

**DOI:** 10.1186/1751-0759-8-4

**Published:** 2014-01-20

**Authors:** Kenji Kawakita, Kaoru Okada

**Affiliations:** 1 Department of Physiology, Meiji University of Integrative Medicine, Hiyoshi-cyo, Nantan-City, Kyoto 629-0392, Japan

**Keywords:** Acupuncture therapy, Action mechanism, Polymodal receptor, Efficacy, Safety, Randomized controlled trials, Systematic review, Psychogenic disorders

## Abstract

Scientific bases for the mechanism of action of acupuncture in the treatment of pain and the pathogenic mechanism of acupuncture points are briefly summarized. The efficacy and safety of acupuncture therapy is discussed based on the results of German clinical trials. A conclusion on the role for acupuncture in the treatment of psychogenic disorders could not be reached.

## Introduction

Acupuncture therapy has been known as a practice associated with Oriental Medicine, and it has recently been identified in the field of Complementary and Alternative Medicine as a potential therapeutic procedure for which there is good scientific evidence [1]. Oriental Medicine is usually characterized as having a unique pathophysiological concept of disease, key components of which are the flow of *qi*, a kind of energy. The absence of a clear distinction between body and soul, known as “mind-soul unity (心身一如)” in Kampo Medicine, is also an important characteristic of Oriental Medicine. Based on this unique concept, psychogenic disorders are understood as conditions caused by the abnormal flow of chi; this understanding is sufficient even when Western medicine is unable, through examination of the body or brain using modern technology, to link the condition to a precise cause. The concept of *qi* and the mechanisms that regulate *qi* are not understood precisely. Nevertheless *qi* it is an important concept in understanding the human being as a self-regulatory organism [2].

Acupuncture therapy originated in China and is often assumed to be a part of Traditional Chinese Medicine (TCM). It has its own system for the diagnosis and treatment of disease. Acupuncture therapy based on TCM is used widely around the world, but other methods of acupuncture exist, and numerous variations of acupuncture therapy have been developed. This is especially true in Japan, where a different form of acupuncture has been developed that uses fine needles with shallow insertion without *de-qi*, which refers to a sensation of numbness, distension, or tingling at the needling site which radiates along the corresponding meridian that is considered an essential feature of acupuncture therapy according to TCM [3]. The characteristics of Japanese acupuncture procedures, shallow needling without de-qi sensation, are now the focus of clinical research, in consideration of the sham interventions used.

This review briefly summarizes what is known concerning the mechanism of action underlying the effects of acupuncture, the current understanding of the so-called acupuncture points, and recent evidence for the clinical efficacy and safety of acupuncture in the treatment of chronic pain (focused on lower back pain). In addition, recent studies of acupuncture therapy in several psychogenic disorders (anxiety; depression; and posttraumatic stress disorder, PTSD) are introduced.

### Action mechanisms of acupuncture therapy

**Figure 1:**
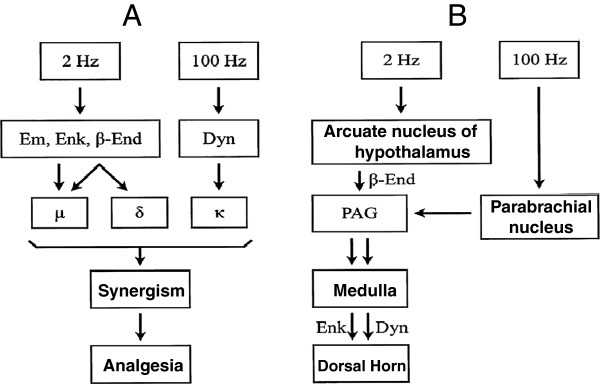
**Schematic illustrations of endogenous opioid-mediated electro-acupuncture analgesia (EAA). (A)** EA at 2 Hz or 100 Hz releases different endogenous opioids, which act on different opioid receptors to induce synergic analgesic effects. **(B)** Impulses elicited by EA at 2 Hz or 100 Hz project to different brain areas and induce analgesic effects through descending inhibition. Em: endomorphine; Enk: encephalin: β-End: β-endorphin; Dyn: dynorphin; μ: mu-opioid receptors; δ: delta opioid receptor; κ: kappa opioid receptor; PAG: periaqueductal grey matter. Modified from Han 2003 reference [7].

Scientific research into the mechanism of action of acupuncture began around 1950 when an important pharmacological study was published by a group at Peking University. They demonstrated that an induction time of 15 to 20 minutes is required for the development of an analgesic effect and proposed the participation of chemical substances in the analgesic actions of acupuncture [4]. Endogenous opioid peptides (EOPs) were considered major candidates for a role in acupuncture’s action, as electro-acupuncture analgesia (EAA) is antagonized by the opioid receptor antagonist naloxone [5]. Furthermore, an increase in EOPs in plasma or cerebrospinal fluid (CSF) has been observed in humans following EAA [6]. Han’s group in Peking University demonstrated a frequency-dependent involvement of different EOPs in electro-acupuncture- (EA)-induced analgesia, using various methods to identify the different opioid receptors and their endogenous agonists. Based on several lines of evidence, Han concluded that low-frequency (2 Hz) EAA is induced by the activation of mu- and delta-opioid receptors via the release of enkephalin, beta-endorphin, and endomorphin in supraspinal CNS regions, whereas the effects of high-frequency (100 Hz) EAA involve the actions of dynorphin on kappa opioid receptors in the spinal cord [7]. Figure 1 summarizes the mechanisms of EAA at different frequencies.

It should be noted, however, that patients treated for symptoms of pain in acupuncture clinics usually obtain pain relief immediately, with no induction time, and this may be due to gentle manipulation of the fine needle. Therefore, different mechanisms may need to be considered to explain the immediate effects of acupuncture. One possible mechanism to explain the immediate suppression of pain by conditioning stimulation is known as diffuse noxious inhibitory controls (DNIC), first reported by Le Bars et al. [8] based on studies in anesthetized rats. According to DNIC, a noxious stimulus applied to any region of the body can induce immediate suppression of pain transmission in neurons of the trigeminal caudalis and/or the spinal dorsal horn. Bing et al. [9] demonstrated clearly that manual acupuncture to the Zusanli (ST36) can induce DNIC-like suppression and that the effect is partially antagonized by naloxone.

The mechanism of DNIC requires activation of thin afferent fibers (A-delta and C fibers), as these are activated by a noxious pinch, immersion into a hot-water bath, or injection of analgesic substances into muscle [10]. Therefore, afferent DNIC input are derived from nociceptors responsive to mechanical, thermal, and chemical stimuli. These receptors are distributed in skin, muscle, and viscera throughout the entire body. The characteristics of these afferent inputs are very similar to those of polymodal receptors (PMR) [11].

### Understanding so-called acupuncture points

Acupuncture points are considered the essential components of acupuncture therapy for diagnosis and treatment. Unfortunately, despite numerous studies undertaken to define the significance of acupuncture points from anatomical or histological perspective, no clear evidence of their existence has been established. On the other hand, some physiological characteristics of acupuncture points such as tenderness and palpable hardenings are considered as sensitization of nociceptors and their effector functions at least in part.

The application of acupuncture induces in the patient a specific sensation called *de-qi*. This *de-qi* sensation is considered essential for effective acupuncture; however, the receptors and afferent fibers responsible for *de-qi* have not been identified. Injection of local anesthetics into a muscle nerve bundle innervating the Ho-ku (LI4) acupuncture points completely abolished the *de-qi* induced by acupuncture manipulation [12].

**Figure 2:**
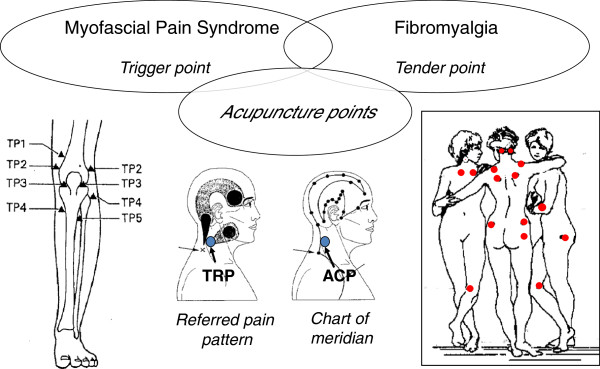
**Close relationships among trigger points, acupuncture points, and tender points.** Trigger points in patients with myofascial pain syndrome appear close to those of acupuncture points. TP1-5 around the knee joints (bottom left figure) were very similar to acupuncture-point loci. The diagnostic tender points for fibromyalgia patients (dots in the bottom right figure) were also very similar to acupuncture-point loci. Referred pain phenomena might be a possible explanation of meridian (center of bottom figures).

Chinese traditional textbooks describe the *ah-shi* point, the point that is hit by the inserted needle to evoke a vocal reaction to pain, as a type of acupuncture point. That is, tenderness is one of the physiological characters of acupuncture points. Our previous survey clearly demonstrated that tender points, as well as acupuncture points, are frequently used in clinical situations by Japanese acupuncturists [13]. Trigger points identified in patients with myofascia1 pain syndrome (MPS) are characterized by tenderness at the restricted point on the palpable band, and their activation can reproduce specific referred pain patterns and phenomena similar to those suffered by the patient [14]. Melzack et al. [15] reported the locations of trigger points were in concordance with those of the acupuncture points. On the other hand, the distribution of tender points in patients with fibromyalgia was also quite similar to that of acupuncture points [16]. The concept of trigger points was established from modern Western medical treatment of pain, apart from any knowledge of TCM. Figure 2 shows the close relationship between trigger points, tender points, and acupuncture points. These similarities suggest that a common pathophysiological mechanism may exist.

Pathophysiological mechanisms for the formation of tender loci have not been clearly established. A possible mechanism may be the presence of sensitized nociceptors at these loci. Itoh et al. [17] developed an experimental model of myofascial trigger points by repeated eccentric contractions of the forearm muscle, which induced delayed-onset muscle soreness. A localized tender locus was formed on the palpable band, and pressure applied to the locus induced a specific referred pain pattern similar to that observed in MPS patients. Important information regarding the pathogenesis of myofascial trigger points was found in human subjects using microdialysis. Shah et al. [18] demonstrated regions rich in protons, bradykinin, inflammatory cytokines (IL-6, TNF-alpha), and the peptidergic neurotransmitters substance P and calcitonin gene-related peptide (CGRP) under the active trigger points. These data clearly indicate the presence of local inflammation under the trigger points.

Substance P and CGRP are known as neurotransmitters of the PMR, and activation of the PMR could release these neuropeptides from nerve terminals to induce neurogenic inflammation. The fact that both acupuncture and stimulation with moxibustion can provoke flare and wheal responses strongly suggests the participation of the PMR in the peripheral mechanism of action for acupuncture [11,19].

### Efficacy of acupuncture therapy: evidence of recent clinical trials

Numerous clinical trials of acupuncture have been conducted, and as the quality of these trials has improved, so has the quality of the information supporting evidence-based acupuncture therapy. Among these acupuncture trials, the German mega trials marked a new era in acupuncture research. The details of the German trials called ART, ARC, COMP, and GERAC were presented by Cumming [20], and we refer the reader to his review for further information regarding the German Acupuncture Trials. Furthermore, numerous systematic reviews and meta-analyses of acupuncture for the treatment of chronic musculoskeletal pain have also been published, and later revised and updated [21-23].

In this section, the GERAC and ART projects for low back pain were selected to introduce the results obtained in high quality clinical trials [24,25]. The GERAC projects were designed as RCTs of three parallel arms with real acupuncture, sham acupuncture, and standard care (guideline-based treatment). The ART project also featured RCTs with three parallel arms of real acupuncture, sham acupuncture, and a waitlist. Real acupuncture employed deep needling to classical acupuncture points with needle manipulation to produce the de-qi sensation. Ten treatments were given over a 6-week period. Sham acupuncture involved superficial needling to standardized non-acupuncture points (outside known acupuncture points). Standard care used best conventional care based on guidelines using multimodal treatment programs including physiotherapy, exercise, and treatment with non-steroidal anti-inflammatory drugs (NSAIDs). The primary outcome measure was response rate after 6 months, defined as improvement of 33% or better on three pain-related items on the Von Korff Chronic Pain Grade Scale (CPGS) questionnaire or improvement of 12% or better on the back-specific Hanover Functional Ability Questionnaire (HFAQ).

**Figure 3:**
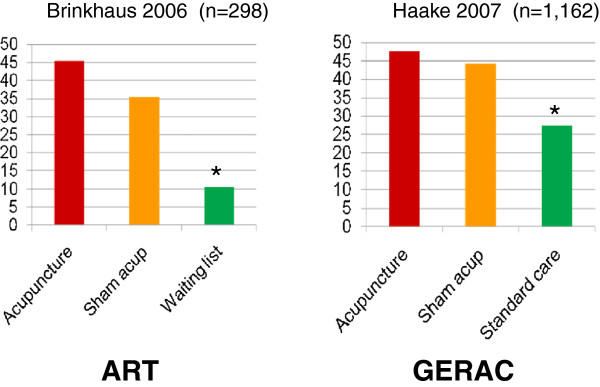
**Results of German acupuncture trials for low back pain.** Both ART and GERAC studies produced similar results. Response rates were significantly higher than waitlist control (ART) and standard care (GERAC), but no difference between real acupuncture and sham (minimal) acupuncture was identified. Cited from Kawakita 2010 reference [25].

Figure 3 summarizes the results of GERAC and ART trials of low back pain. The response rates at 6 months were 47.6% (real acupuncture), 44.2% (sham acupuncture), and 27.4% (standard care). The differences among groups were as follows: acupuncture vs standard care, 20.2% (*P* < 0.001); and minimal acupuncture vs standard care, 16.8% (*P* < 0.001). Compared to standard care, both real and sham acupuncture provided superior pain relief. As shown in Figure 3, larger differences between the real and sham acupuncture groups vs the waitlist group were observed.

Chronic low back pain is usually classified as a type of musculoskeletal disorder, but its underlying cause involves more than simple tissue injury of the lower back. It is a more complex phenomenon that includes neural plasticity and changes in pain-transmission circuitry as well as psychogenic problems. The unexpectedly low correlation between the physical disorder and the clinical presentation of low back pain (r = 0.27) [26] and the failure of the majority of the 200 patients with chronic low back pain in rehabilitation programs to meet the diagnostic criteria for at least part of the DSM-III-R Axis I disorder [27] suggest the existence of psychogenic factors as essential components of chronic low back pain. The term *psychogenic low back pain* was proposed to stress the importance of psychogenic factors in such patients [28].

The results from the GERAC project on low back pain clearly demonstrate that acupuncture (real and sham) provided therapeutic effects superior to those from guideline-based standard care. In the GERAC project, physiotherapy and NSAIDs were the primary interventions for patients in the standard-care group, and no medications for psychological disorders were prepared in the protocol. Therefore, the superior effectiveness of the acupuncture interventions may be, at least in part, the result of the therapeutic effects of acupuncture on the psychogenetic aspects of lower back pain.

In the GERAC and ART projects, no statistical difference was detected between the real and sham groups. The sham acupuncture used in the GERAC and ART projects was minimal acupuncture applied to the non-acupuncture points and did not produce a *de-qi* sensation. The fact that minimal acupuncture was more effective than guideline-based standard care strongly suggests that minimal acupuncture is not physiologically inert and cannot be considered merely placebo intervention.

To confirm the specific effects of acupuncture therapy, a well-designed, four-arm RCT of acupuncture was conducted in the USA [29]. Instead of minimal acupuncture using needle insertion, simulated acupuncture using a wooden toothpick to prick the skin was employed as a sham intervention. To investigate the significance of TCM acupuncture procedures, individualized diagnosis and prescription of acupuncture points was used in one arm. Another arm used standardized TCM acupuncture manipulation, and the fourth arm was standard care. Compared with standard care, individualized acupuncture, standardized acupuncture, and simulated acupuncture had beneficial and persisting effects on chronic low back pain. These treatments resulted in clinically meaningful improvements and provided strong and consistent evidence that real acupuncture needling using the Chinese meridian system is no more effective for chronic back pain than various forms of sham acupuncture. Nevertheless, both real and sham acupuncture were superior to standard care.

It should be noted that the majority of acupuncture trials have demonstrated that both real acupuncture and sham acupuncture produce effects that are superior to those from standard care, but no significant difference between real and sham acupuncture have been identified. Minimal acupuncture, as used in the German mega trials, was considered an insufficient form of acupuncture stimulation, because it fails to evoke a *de-qi* sensation and does not use acupuncture points. Simulated acupuncture involving a toothpick to prick the skin seems to be an inert intervention, as it does not penetrate the skin. However, both the minimal and simulated sham interventions are undoubtedly active stimulations, from the perspective of Japanese-style acupuncture.

Japanese acupuncture therapy is characterized by shallow penetration with a fine needle. Press tack needles and intradermal needles are frequently used in clinical situations. No-insertion needles, i.e., using needles to scratch the skin, are also used. The *de-qi* sensation essential for TCM is not usually required. Japanese acupuncture procedures seem to be similar to those used as minimal acupuncture in the German trials. Acupuncture points selected by well-trained Japanese acupuncturists may not be restricted to anatomically defined points. Restricted points are usually identified based on tenderness, local hardening, and other characteristics of the skin and/or subcutaneous tissues.

There are numerous RCTs, systematic reviews, and meta-analyses of acupuncture in chronic-pain patients that show no significant differences between real and sham acupuncture. These results have tended to lead to the conclusion that acupuncture provides no specific effect and is simply a type of strong placebo effect. This conclusion may be based reasonably on the lack of statistically significant differences between different forms of acupuncture and the observation that they are nevertheless more effective than guideline-based standard care.

### Safety of acupuncture therapy

Acupuncture needling can cause serious adverse events when inserted inadequately, as the insertion of the needle induces tissue injury. In the 1980s, cross-infection of the hepatitis B virus through the use of unsterilized acupuncture needles was identified as another serious issue. With increased awareness of the importance of preventing such infections through the use of single-use disposable needles, such infections have been mostly eliminated in acupuncture therapy by professional healthcare providers.

Regarding adverse events related to acupuncture therapy, numerous reliable data are now available from prospective surveys of acupuncture safety conducted in the United Kingdom [30,31], Germany [32,33], and Japan [34]. In a larger-scale German survey of over 9000 German physicians providing 760 000 acupuncture treatments, there were only six reported cases of adverse events such as pneumothorax, exacerbation of depression, acute hypertensive crisis, vasovagal reaction, and asthma attack with hypertension and angina [32]. In another large survey of 2.2 million consecutive acupuncture treatments, adverse events were detected in two patients (pneumothorax and lower limb nerve injury) [33]. On the other hand, there is a known association between NSAID use and internal bleeding or perforated gastro-duodenal ulcers, and a rigorous survey demonstrated that, on average, one in 1200 patients taking NSAIDs for at least two months will die due to gastro-duodenal complications [35]. The evidence available from large-scale prospective surveys indicates that acupuncture therapy is safe, and serious adverse events and deaths caused by acupuncture therapy are rare.

### Acupuncture therapy for psychogenic disorders

For acupuncture therapy to gain acceptance as a treatment for psychogenic disorders, sufficient evidence will be required. It has been well established that systematic reviews of well conducted RCTs and meta-analyses offer the strongest evidence for evidence-based medicine. Regarding acupuncture therapy for psychogenic disorders, systematic reviews of depression, anxiety, and post-traumatic stress disorder (PTSD) have been published, and we introduce brief summaries of their results. The methods for systematic reviews and meta-analyses were theoretically similar among the studies. Systematic searches of references related to the research questions were conducted using various databases, and an adequate number of studies meeting the inclusion criteria were identified. The details of the selected references were further analyzed. When possible, the patient data were pooled for meta-analysis.

The following section briefly summarizes the results of systematic reviews and meta-analyses of clinical trials of acupuncture in the treatment of depression, anxiety, and PTSD.

#### Depression

Zhang et al. [36] conducted a systematic review and meta-analysis of 207 clinical studies in which acupuncture was used to treat depression. They included twenty RCTs of major depressive disorder (MDD, n = 1998) and 15 RCTs of post-stroke depression (PSD, n = 1680) for meta-analysis. The efficacy of acupuncture as a mono-therapy was comparable to antidepressants alone in improving the clinical response and alleviating symptom severity in MDD, but its efficacy was not different from that of sham acupuncture. There was insufficient evidence to support the expectation that acupuncture combined with antidepressants could yield better outcomes than antidepressants alone in treating MDD. Acupuncture was superior to antidepressants and waitlist controls in improving both the response and symptom severity in PSD. The researchers concluded that acupuncture therapy is safe and effective for the treatment of MDD and PSD, and could be considered an alternative option for the treatment of both disorders. The efficacy of acupuncture in other forms of depression remains to be determined.

Ernst et al. [37] conducted a systematic review of systematic reviews, to produce a critical evaluation with a view toward assisting clinical decisions. Eight systematic reviews including seventy-one primary studies were identified. Five of the reviews arrived at positive conclusions, and three did not. All the positive reviews and most of the positive primary studies originated from China. The authors concluded that the effectiveness of acupuncture as a treatment for depression remains unproven.

#### Anxiety and anxiety disorders

Pilkington selected 10 RCTs for systematic review [38]. Four RCTs focused on acupuncture in generalized anxiety disorder or anxiety neurosis, while six focused on anxiety in the perioperative period. In generalized anxiety disorder or anxiety neurosis, all trials reported positive findings, but the reports lacked detailed information regarding methods. Reporting in the studies of perioperative anxiety was generally better. The initial indication was that acupuncture, specifically auricular acupuncture, was more effective than acupuncture at sham points and might be as effective as drug therapy. Positive findings were reported for acupuncture in the treatment of generalized anxiety disorder or anxiety neurosis, but they concluded there was insufficient research evidence to support a firm conclusion. There is some limited evidence in favor of auricular acupuncture for the treatment of perioperative anxiety.

#### PTSD

Kim et al. [39] reported the first systematic review and meta-analysis of acupuncture for the treatment of PTSD. In their systematic review, 136 potentially relevant references were identified, and 16 articles were evaluated. Finally, four RCTs meeting the inclusion criteria were identified. All four studies used a parallel-group design, and two of the four were based on a sample size calculation. The four RCTs evaluated 543 PTSD patients, with a duration of treatment from 1 to 12 weeks.

One high-quality RCT evaluated the effect of acupuncture versus cognitive-behavior therapy (CBT) and a waitlist control. No statistical differences were found between acupuncture and CBT, but acupuncture treatment was statistically superior to waitlist control. The therapeutic effects of acupuncture and CBT on the effects sizes were similar. One RCT evaluated the effect of electro-acupuncture versus oral SSRI treatment, and no statistical differences were found between the groups. The main finding of this review was that acupuncture is effective for PTSD, based on one high-quality RCT and a meta-analysis. It showed that the effectiveness of acupuncture was statistically superior to waitlist control, although no statistical difference was found between the effectiveness of acupuncture and CBT. The meta-analysis showed that acupuncture plus moxibustion was superior to oral SSRI treatment for PTSD. The results, however, should be evaluated carefully, as the meta-analysis was based on one medium-quality and one low-quality RCT. The conclusion they reached was that the evidence for the effectiveness of acupuncture for PTSD is encouraging but not cogent, because the number of studies included in the meta-analysis, two RCTs, was too small to verify its efficacy.

## Conclusions

Acupuncture therapy has long been a well known part of Traditional Chinese Medicine. Recent advances in basic studies have demonstrated the mechanistic basis for so-called acupuncture points, as well as describing their functional characteristics. Increases in the number of large-scale clinical trials and systematic reviews of acupuncture in the treatment of chronic musculoskeletal pain have clearly demonstrated the efficacy and safety of acupuncture therapy. On the other hand, the number of clinical trials examining the efficacy of acupuncture in the treatment of psychogenic disorders has increased. However, the quality of the studies has been relatively poor and the sample sizes insufficient. Although positive results for acupuncture therapy in psychogenic disorders have been observed in several RCTs, recent systematic reviews and meta-analyses have not offered any firm conclusions. Further clinical study using rigorous experimental design and sufficient sample sizes will be required.

## Competing interests

The authors declare that they have no competing interests.

## Authors’ contributions

The manuscript was written and revised by KK (corresponding author). KO performed background literature surveys, provided citations, and checked the final format for citations and references. Both authors read and approved the final manuscript.
